# Optimal large language models to screen citations for systematic reviews

**DOI:** 10.1017/rsm.2025.10014

**Published:** 2025-06-23

**Authors:** Takehiko Oami, Yohei Okada, Taka-aki Nakada

**Affiliations:** 1 Department of Emergency and Critical Care Medicine, Chiba University Graduate School of Medicine, Chiba, Japan; 2 Department of Preventive Services, Kyoto University Graduate School of Medicine, Kyoto, Japan; 3 Pre-hospital and Emergency Research Centre, Health Services and Systems Research, Duke-NUS Medical School, National University of Singapore Singapore

**Keywords:** citation screening, clinical practice guidelines, generative AI, large language models, systematic review

## Abstract

Recent studies highlight the potential of large language models (LLMs) in citation screening for systematic reviews; however, the efficiency of individual LLMs for this application remains unclear. This study aimed to compare accuracy, time-related efficiency, cost, and consistency across four LLMs—GPT-4o, Gemini 1.5 Pro, Claude 3.5 Sonnet, and Llama 3.3 70B—for literature screening tasks. The models screened for clinical questions from the Japanese Clinical Practice Guidelines for the Management of Sepsis and Septic Shock 2024. Sensitivity and specificity were calculated for each model based on conventional citation screening results for qualitative assessment. We also recorded the time and cost of screening and assessed consistency to verify reproducibility. A *post hoc* analysis explored whether integrating outputs from multiple models could enhance screening accuracy. GPT-4o and Llama 3.3 70B achieved high specificity but lower sensitivity, while Gemini 1.5 Pro and Claude 3.5 Sonnet exhibited higher sensitivity at the cost of lower specificity. Citation screening times and costs varied, with GPT-4o being the fastest and Llama 3.3 70B the most cost-effective. Consistency was comparable among the models. An ensemble approach combining model outputs improved sensitivity but increased the number of false positives, requiring additional review effort. Each model demonstrated distinct strengths, effectively streamlining citation screening by saving time and reducing workload. However, reviewing false positives remains a challenge. Combining models may enhance sensitivity, indicating the potential of LLMs to optimize systematic review workflows.

## Highlights

### What is already known?

- Large language models (LLMs) have shown promise in citation screening for systematic reviews, but direct comparisons of their performance, particularly in terms of accuracy, efficiency, and cost, are lacking.
- Understanding these differences is crucial for optimizing systematic review workflows.

### What is new?

- This study provides a comparative analysis of four LLMs—GPT-4o, Gemini 1.5 Pro, Claude 3.5 Sonnet, and Llama 3.3 70B in literature screening tasks, revealing distinct trade-offs between sensitivity and specificity.
- The results suggest that while GPT-4o and Llama 3.3 70B offer higher specificity, Gemini 1.5 Pro and Claude 3.5 Sonnet deliver better sensitivity.
- An ensemble approach of these models increases sensitivity at the cost of specificity, indicating potential synergies.

### Potential impact for RSM readers

- This study may guide researchers and practitioners in selecting appropriate LLMs for literature screening tasks based on their specific needs for accuracy, efficiency, and cost-effectiveness.
- The findings suggest that strategically combining outputs from multiple LLMs can enhance screening accuracy, potentially streamlining systematic review processes.

## 1 Background

Systematic reviews involve query formulation, citation screening, qualitative assessments, and meta-analyses. They play a pivotal role in aggregating and synthesizing the latest scientific evidence to better inform the development of new guidelines and standard practices.^1^ ^,^ ^2^ Manual citation screening, a labor-intensive and time-consuming task typically involved in the systematic review process, often results in the introduction of human error and time delays.^3^ ^-^ ^5^ Although recent advances in machine learning have streamlined these tasks,^6^ ^-^ ^11^ balancing efficiency with accuracy remains a significant challenge.^11^ ^-^ ^13^

Machine learning approaches typically rely on training data and heuristic rules to conduct citation screening, thereby human workload is needed to complete the process.^14^ Additionally, previous studies have highlighted potential limitations in semi-automatic approaches, such as performance variability due to data-algorithm interactions.^15^ ^,^ ^16^ These challenges, however, may be addressed with the emergence of large language models (LLMs), which are pre-trained by model developers, eliminating the need for users to provide prior input. Furthermore, LLM-assisted citation screening does not require predefined rules determining when to discontinue manual screening conducted for additional learning, as LLMs can execute comprehensive screening.^17^ ^,^ ^18^ Recent studies have explored the potential of LLMs to enhance the efficiency of complex citation screening tasks in healthcare by leveraging the advanced natural language-processing and task-execution capabilities of these models.^19^ ^,^ ^20^

Recent LLMs such as GPT, Gemini, Claude, and Llama have shown promising potential for understanding and generating natural language-based responses, which may streamline the citation screening process.^21^ ^,^ ^22^ Consequently, studies have proposed using LLMs as “second reviewers” to assist with manual citation screening, by distinguishing between relevant and irrelevant literature.^23^ ^,^ ^24^ Identification of potentially relevant studies leads to reducing the screening workload and enhancing efficiency in systematic review processes. In our previous study, GPT-4 Turbo demonstrated a high specificity for this application; however, its sensitivity remained insufficient.^25^ As sufficient sensitivity has been defined as achieving a recall rate of at least 95%, such accuracy with minimal risk of missing relevant studies would be needed in this area.^5^ ^,^ ^6^ ^,^ ^16^ As the performance of LLMs continues to evolve, using the latest models or integrating multiple LLMs may represent promising strategies for enhancing their accuracy and efficiency related to citation screening. While existing studies have tested LLMs for citation screening, most of these studies have focused on a single LLM.^24^ ^-^ ^28^ The performance of LLMs potentially accounts for differences in dataset characteristics, variations in prompt design and configuration, and inherent limitations in the model’s training data and architecture.^15^ ^,^ ^16^ Consequently, there remains a gap in comprehensive studies comparing the performance of multiple LLMs or investigating their combined application for citation screening.

We therefore hypothesized that the optimal LLMs for screening citations could be identified based on their inherent capabilities to process natural language, identify patterns, and generate outputs based on training data. This study aimed to evaluate and compare the performance of four currently popular LLMs—GPT-4o, Gemini 1.5 Pro, Claude 3.5 Sonnet, and Llama 3.3 70B—with regard to screening literature based on titles and abstracts using clinical questions (CQs) for systematic reviews and meta-analyses in the development of the Japanese Clinical Practice Guidelines for the Management of Sepsis and Septic Shock (J-SSCG) 2024.

## 2 Methods

### 2.1 Study design and settings

We conducted a prospective study to evaluate the performance of LLMs for citation screening. To ensure the transparency and accessibility of our methods, we uploaded our detailed review protocol to the medRxiv preprint platform (https://www.medrxiv.org/content/10.1101/2024.06.26.24309513v1). The study was registered in the University Hospital Medical Information Network clinical trials registry (UMIN000054783). Any deviations from the protocol are detailed in the Supplementary Appendix. We adhered to the guidelines outlined in the Standards for Reporting of Diagnostic Accuracy.^29^ The source code for this study is publicly available in the GitHub repository (https://github.com/seveneleven711thanks39/llm-assisted_citation_screening.git).

### 2.2 Clinical questions in the J-SSCG

We evaluated the accuracy of the four LLMs using CQs for systematic reviews and meta-analyses from J-SSCG 2024. These guidelines were developed by the Japanese Society of Intensive Care Medicine and the Japanese Association for Acute Medicine to guide the management of sepsis and septic shock in Japanese healthcare settings. A published version of J-SSCG 2024 is available on the following websites: https://www.jsicm.org/en/ and https://www.jaam.jp/english/english-top.html. Specifically, the Japanese version of J-SSCG 2024 was published on December 25, 2024, and the English version was published on March 14, 2025.^30^

We used the same five CQs selected in our previous related study to address the issues highlighted in the prior work (Table 1).^13^ These CQs were newly outlined by the working group members and the guideline committee during the guideline development process. As the selected CQs belonged to the same domain, there is a potential for reference overlap between the CQs, which could lead to data dependency issues. Extensive literature searches were conducted across several databases, including CENTRAL, PubMed, and Ichushi-Web. The working group meticulously formulated search strategies to achieve comprehensive coverage of all relevant studies based on the patient/population/problem, intervention, comparison, and study design of each CQ. The search was limited to studies published in both Japanese and English. For the J-SSCG 2024, EndNote (Clarivate Analytics, Philadelphia, PA, USA) was used as a citation management software.^31^ This software was instrumental in downloading, organizing, and eliminating duplicate entries from the titles and abstracts collected during the literature search. The whole conventional citation screening process has been conducted by the systematic review members of J-SSCG 2024, and the data of the screening process were provided to our research team (J-SSCG 2024, AI application taskforce). This data sharing and collaboration were organized by the authors (T.O., Y.O., and T.N.). T.N. chaired the J-SSCG 2024 special committee. The members of the conventional screening team are listed in the Acknowledgements section.

**Table 1:** List of the patient/population/problem, intervention, comparison, and study design of the selected CQs

	Patient, population, problem	Intervention	Comparison	Study design
CQ1	Adult patients (18 years or older) diagnosed with, or suspected of having, an infection, bacteremia, or sepsis	Balanced crystalloid administration	0.9% sodium chloride administration	Randomized controlled trial
CQ2	Adult patients (18 years or older) with sepsis or suspected sepsis, an infection, bacteremia, or patients admitted to ICU	Targeting a higher mean arterial pressure	Targeting a lower mean arterial pressure	Randomized controlled trial
CQ3	Adult patients (18 years or older) with sepsis presenting with severe metabolic acidosis or patients admitted to ICU	Sodium bicarbonate administration	No sodium bicarbonate administration	Randomized controlled trial
CQ4	Adult patients (18 years or older) with sepsis or septic shock	Usual care with at least one of the following tissue perfusion parameters: lactate/lactate clearance, capillary refill time, ScvO_2_/SvO_2_, and P(v-a) CO_2_/C (a-v) O_2_	Usual care with different parameters mentioned in the interventional group or standard care without the utilization of any specific tissue perfusion parameters	Randomized controlled trial
CQ5	Adult patients (18 years or older) with sepsis, sepsis-induced hypotension, or septic shock	Restrictive fluid management aiming to reduce the amount of fluid therapy for up to 24 h	Conventional fluid management or non-restrictive fluid management defined by authors	Randomized controlled trial

Abbreviations: CQ, clinical question; ICU, intensive care unit.

### 2.3 Conventional citation screening

The conventional citation screening is composed of title/abstract and full-text screening. The members of J-SSCG 2024 transferred the files managed in EndNote to Rayyan, a software specifically designed for systematic reviews.^32^ The screening process involved two independent reviewers who individually assessed the titles and abstracts of each study. Disagreements were resolved through discussion or, when necessary, by consulting a third neutral reviewer. Following title/abstract screening, the members of J-SSCG 2024 performed full-text screening and determined the inclusion of qualitative assessment in the systematic review processes. We used the results of manual citation screening as a standard reference to assess accuracy. The characteristics of the reviewers for the five CQs were collected, including age, sex, professional role, educational qualifications, field of expertise, years of clinical experience, and the total number of systematic reviews published. The authors of the current study (T.O., Y.O., and T.N.) were not directly involved in the conventional citation screening process. The conventional systematic review process was completed when the performance of the LLMs was evaluated separately. To maintain the integrity of the assessment, the final determination of relevance or irrelevance from the conventional citation screening was concealed from the LLMs.

### 2.4 LLMs

Four LLMs were evaluated: GPT-4o (OpenAI, San Francisco, CA, USA), Gemini 1.5 Pro (Alphabet, Inc., Mountain View, CA, USA), Claude 3.5 Sonnet (Anthropic, San Francisco, CA, USA), and Llama 3.3 70B (Meta, Menlo Park, CA, USA), which were released on May 13, 2024; May 23, 2024; June 21, 2024; and December 6, 2024, respectively. Our objectives were to compare these models in terms of accuracy, time efficiency, cost, and consistency. After importing the dataset from the citation management tool following the conventional method, we connected it to the application programming interface (API) of each LLM using Pandas version 1.0.5 in Python version 3.9.0. These publicly available APIs allowed us to interface more easily with each of the LLMs. To conduct the LLM-assisted citation screening, we used a command prompt that enabled the LLMs to automatically execute the citation screening process described in a previous report as follows^25^:

You are conducting a systematic review and meta-analysis, focusing on a specific area of medical research. Your task is to evaluate research studies and determine whether they should be included in your review. To do this, each study must meet the following criteria :

Target Patients: Adult patients (18 years or older) diagnosed with, or suspected of having, an infection, bacteremia, or sepsis.

Intervention: The study investigates the effects of balanced crystalloid administration.

Comparison: The study compares the above intervention with 0.9% sodium chloride administration.

Study Design: The study must be a randomized controlled trial.

Additionally, any study protocol that meets these criteria should also be included.

However, you should exclude studies in the following cases :

The study does not meet all of the above eligibility criteria.

The study’s design is not a randomized controlled trial. Examples of unacceptable designs include case reports, observational studies, systematic reviews, review articles, animal experiments, letters to editors, and textbooks.

After reading the title and abstract of a study, you will decide whether to include or exclude it based on these criteria. Please answer with include or exclude only.

Title: ----------------------

Abstract

-------------------------------------------------------------------------------------------------------

For each query, we strictly adhered to the same phrases outlined in the framework of the CQs formulated by the J-SSCG 2024 members for conventional citation screening (Table 1). The screening process was repeated in triplicate to ensure reproducibility. In the replicate process, the exact same prompt, same data, and same conditions were used each time. We used the results from the first round as representative data because, in practical citation screening workflows, the initial evaluation is typically the primary reference point.

In the LLM-assisted citation screening process, decisions regarding inclusion or exclusion were made without relying on training data, based on the patient/population/problem, intervention, comparison, and study design of each selected CQ.^33^ At the end of each session, the decisions recorded in the output file were downloaded and then reviewed. The LLM-assisted citation screening with GPT-4o, Gemini 1.5 Pro, and Claude 3.5 Sonnet was conducted between June 26 and July 18, 2024, while the screening with Llama 3.3 70B was performed between January 24 and 26, 2025. We performed the citation screening using a 14-inch MacBook Pro (2021) equipped with the Apple M1 Max chip and 32 GB of RAM.

### 2.5 Performance measures

We collected and analyzed the following variables for the primary and secondary analysis.

Accuracy metrics: sensitivity and specificity were calculated based on the number of references identified as “relevant” by each LLM and the number of references identified as “irrelevant” by each LLM. False positives are studies identified as “relevant” by the LLM but determined to be “irrelevant” during the manual review, while false negatives are studies deemed “irrelevant” by the LLM but found to be “relevant” upon the conventional screening. For the primary analysis, the list of included studies for qualitative assessment using the conventional method served as the standard reference. Similarly, for the secondary analysis, the standard reference was the list of studies included after title and abstract screening using the conventional method.

Processing time: the time required for screening 100 studies by each LLM as described in our previous study.^13^ ^,^ ^25^ Briefly, the processing time measurement began when the LLM received the complete input prompt, and ended when the LLM completed its screening output. This includes the time taken for the LLM to process the data and generate relevance decisions for all citations.

Cost: the total costs associated with API usage, calculated using a usage-based pricing model. We measured the difference between the API usage cost recorded just before the start of the review process for each CQ and the cost recorded immediately after the review process was completed.

Consistency: the following formula was used to calculate the consistency to assess the variability in the repeated screening results of the same CQs across different rounds:

Consistency rate = (number of agreements on inclusion + number of agreements on exclusion)/total number of citations.

### 2.6 Statistical analysis

To evaluate and compare the accuracies of the LLMs, we counted the citations they correctly identified as “relevant” and calculated the sensitivity and specificity for each with a 95% confidence interval (CI). Our primary analysis used the results of the manual full-text screening for qualitative assessment. The secondary analysis was based on the results of the title and abstract reviews from the conventional screening. We used meta-analysis methods to calculate the integrated sensitivity and specificity across different CQs separately in the primary and secondary analyses, following the guidelines outlined in the *Cochrane Handbook*.^34^ By using meta-analysis, we ensured that the contribution of each CQ was weighted according to its sample size, providing a more accurate and reliable estimate of performance. A random-effects model was applied to manage variance within and across studies.^35^ We assessed the heterogeneity of the CQs by visual inspection, as suggested by the *Cochrane Handbook*.^34^ The “meta” package (https://cran.r-project.org/web/packages/meta/meta.pdf) in R version 4.1.2 (R Foundation for Statistical Computing) was used for the meta-analysis. The CIs for sensitivity and specificity were calculated according to the “Clopper–Pearson interval” as a default setting.^36^

To assess time efficiency and cost, we compared the temporal durations of the systematic review sessions and the costs associated with the LLM-assisted citation screenings, using APIs, across all CQs between the models. We also performed a final “LLM ensemble” method, which consisted of a *post hoc* analysis examining the impact of combining the results from the four LLMs to potentially further enhance the robustness of the citation screening process.^37^ To maximize sensitivity, we aggregated the individual decisions of each LLM and counted the number of publications identified as “relevant” by any of them. Continuous data are presented as medians and interquartile ranges. GraphPad Prism 10 (GraphPad Software, San Diego, CA, USA) was used for all statistical analyses.

## 3 Results

### 3.1 Conventional citation screening

**Figure 1:**
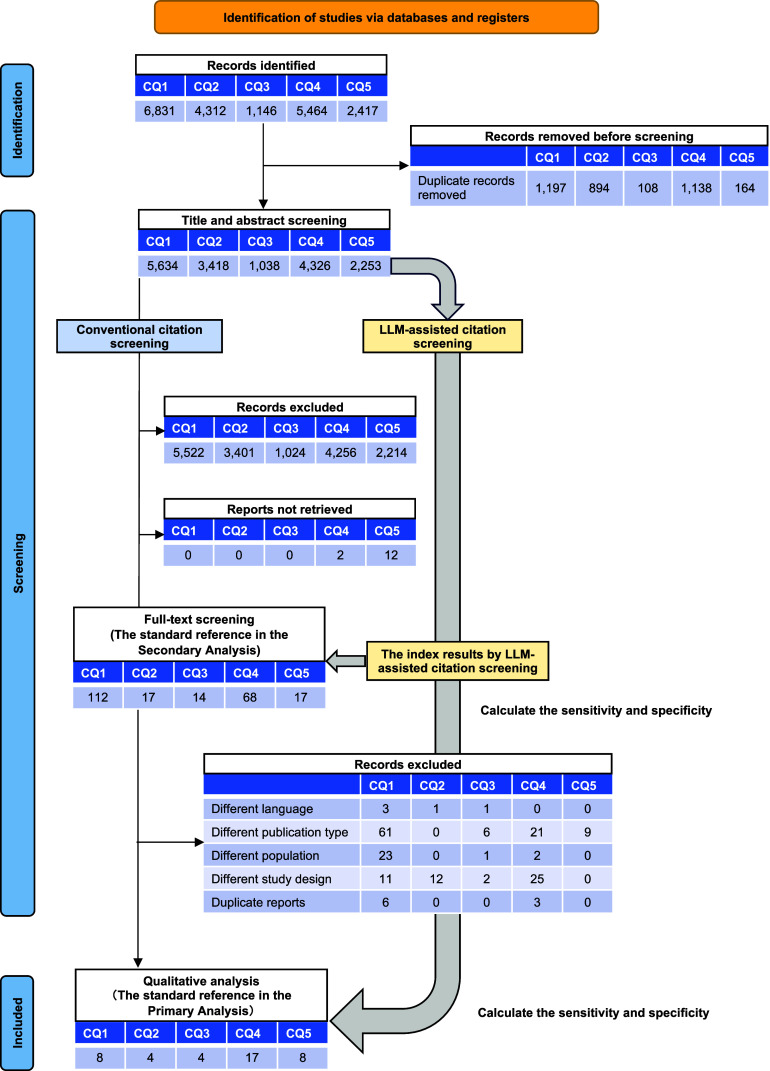
Schematic overview of systematic review between large language model (LLM)-assisted citation screening and the conventional method. Flowchart of the systematic review process: identification, title and abstract screening, and full-text screening. This figure also illustrates the timing of accuracy statistics for LLM-assisted citation screening in the primary and secondary analyses. CQ: clinical question.

In the development of J-SSCG 2024, 18 reviewers performed the conventional citation screening for the five CQs. The majority of the reviewers had medical degrees (95%), while 83.3% had no prior experience with systematic review publications (Supplementary Table S1). The manual citation screening process, based on titles and abstracts, selected 112 of 5,634 publications (2.0%) on CQ1, 17 of 3,418 (0.5%) on CQ2, 14 of 1,038 (1.3%) on CQ3, 70 of 4,326 (1.6%) on CQ4, and 39 of 2,253 (1.7%) on CQ5. Subsequent full-text screening selected a total of 41 publications for qualitative analysis: 8 from CQ1 (0.14%), 4 from CQ2 (0.12%), 4 from CQ3 (0.39%), 17 from CQ4 (0.39%), and 8 from CQ5 (0.36%) (Figure 1).

### 3.2 Literature selection process in LLM-assisted citation screening

During the LLM-assisted citation screening process, the GPT-4o citation screening resulted in 6 publications for CQ1, 3 for CQ2, 4 for CQ3, 17 for CQ4, and 7 for CQ5 being incorporated into the qualitative analysis. The Gemini 1.5 Pro and Claude 3.5 Sonnet screenings selected 8 publications for CQ1, 4 for CQ2, 4 for CQ3, 17 for CQ4, and 8 for CQ5. The Llama 3.3 70B screening included 6 publications for CQ1, 4 for CQ2, 4 for CQ3, 17 for CQ4, and 8 for CQ5 in the final results for the qualitative assessment in the systematic review processes (Figure 1 and Supplementary Tables S2 and S3).

### 3.3 Accuracy of the LLM-assisted citation screening for literature review

**Figure 2:**
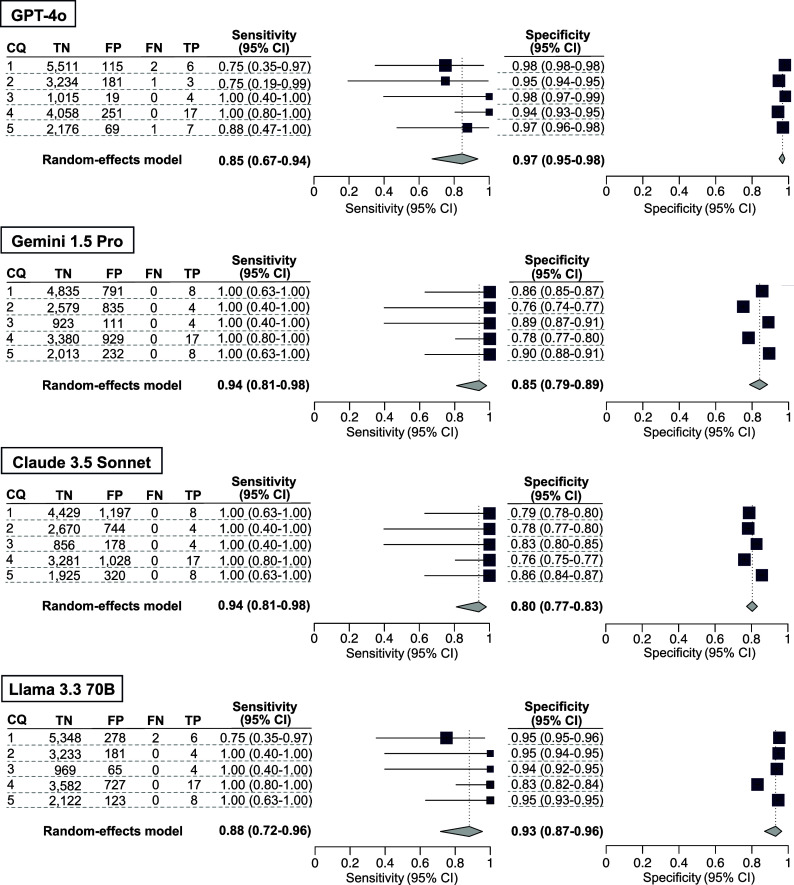
Comparison of four large language models in terms of the accuracy of citation screening: primary analysis. Our primary analysis used the results of the included publications for qualitative assessment, using the conventional method as the standard reference. The individual sensitivity and specificity for each clinical question (CQ) and the integrated sensitivity values across CQs 1–5 were compared among GPT-4o, Gemini 1.5 Pro, Claude 3.5 Sonnet, and Llama 3.3 70B using confidence intervals.

In the primary analysis, the integrated sensitivity and specificity values (respectively) among the three models for the five CQs were 0.85 [0.67–0.94] and 0.97 [0.95–0.98] for GPT-4o, 0.94 [0.81–0.98] and 0.85 [0.79–0.89] for Gemini 1.5 Pro, 0.94 [0.81–0.98] and 0.80 [0.77–0.83] for Claude 3.5 Sonnet, and 0.88 [0.72–0.96] and 0.93 [0.87–0.96] for Llama 3.3 70B (Figure 2 and Supplementary Table S4).

**Figure 3:**
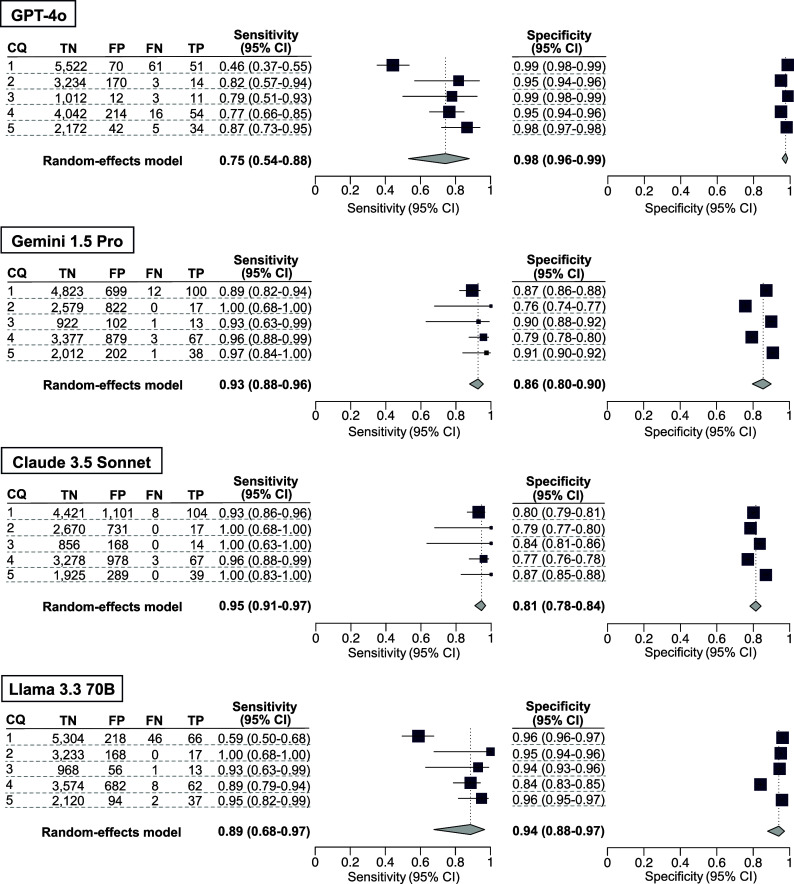
Comparison of four large language models in terms of the accuracy of citation screening: secondary analysis. Our secondary analysis used the results of the included publications for a full-text screening session, using the conventional method as the standard reference. The individual sensitivity and specificity for each clinical question (CQ) and the integrated sensitivity values across CQs 1–5 were compared among GPT-4o, Gemini 1.5 Pro, Claude 3.5 Sonnet, and Llama 3.3 70B using confidence intervals.

In our secondary analysis, the integrated sensitivity and specificity values, respectively (at the 95% CI), for the five CQs using GPT-4o, Gemini 1.5 Pro, Claude 3.5 Sonnet, and Llama 3.3 70B were 0.75 [0.54–0.88] and 0.98 [0.96–0.99], 0.93 [0.88–0.96] and 0.86 [0.80–0.90], 0.95 [0.91–0.97] and 0.81 [0.78–0.84], and 0.89 [0.68–0.97] and 0.94 [0.88–0.97] (Figure 2 and Supplementary Table S5). GPT-4o and Llama 3.3 70B exhibited marked variability in sensitivity and consistent specificity, whereas Gemini 1.5 Pro and Claude 3.5 Sonnet demonstrated consistent sensitivity but notable variability in specificity. The number of true-positive, true-negative, false-positive, and false-negative results are listed in Figures 2 and 3.

### 3.4 Comparison of overall citation screening time, cost for 100 studies, and consistency across the LLMs

**Figure 4:**
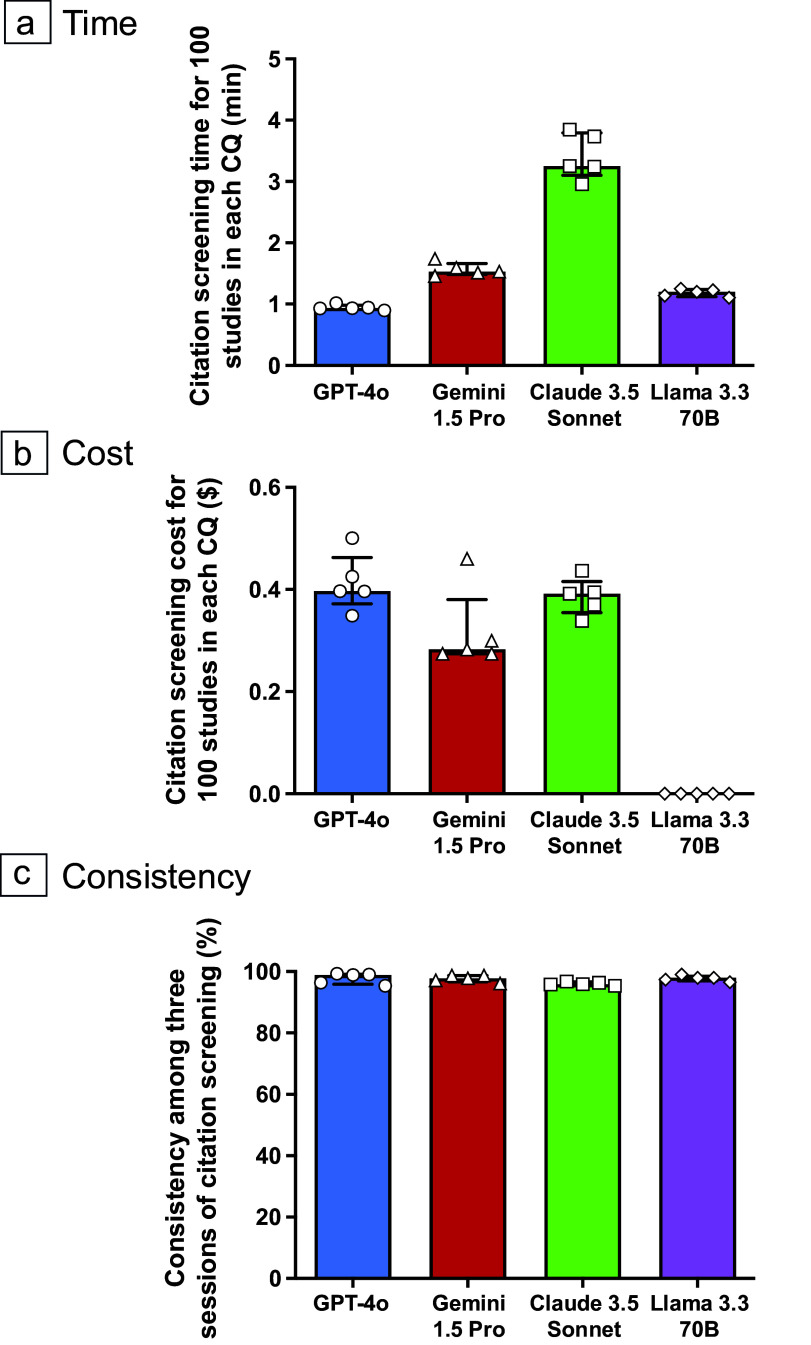
Comparison of citation screening time, cost for 100 studies, and consistency among the four large language models (LLMs). The times needed to process 100 studies, at the 95% confidence interval, using the GPT-4o, Gemini 1.5 Pro, Claude 3.5 Sonnet, and Llama 3.3 70B LLMs were 0.93 [0.92–0.98] min, 1.53 [1.49–1.74] min, 3.25 [3.10–3.79] min, and 1.20 [1.12–1.24] min, respectively (a). The overall citation screening costs for 100 studies (at the 95% confidence interval) for GPT-4o, Gemini 1.5 Pro, Claude 3.5 Sonnet, and Llama 3.3 70B were $0.41 [0.34–0.48], $0.32 [0.22–0.42], $0.39 [0.34–0.43], and $0 [0–0], respectively (b). Consistency rates were calculated using the number of agreements on inclusion or exclusion, and the total number of citations between the three sessions of citation screening. The results for GPT-4o, Gemini 1.5 Pro, Claude 3.5 Sonnet, and Llama 3.3 70B ranged from 95.4% to 99.3%, 96.1% to 98.7%, 95.4% to 96.8%, and 96.5% to 99.1%, respectively (c).

The overall citation screening times for 100 studies (at the 95% CI) using GPT-4o, Gemini 1.5 Pro, Claude 3.5 Sonnet, and Llama 3.3 70B were 0.93 [0.92–0.98] min, 1.53 [1.49–1.74] min, 3.25 [3.10–3.79] min, and 1.20 [1.12–1.24] min, respectively (Figure 4 and Supplementary Table S6). Additionally, the overall citation screening costs for 100 studies using GPT-4o, Gemini 1.5 Pro, Claude 3.5 Sonnet, and Llama 3.3 70B (at the 95% CI) were $0.40 [0.37–0.46], $0.28 [0.27–0.38], $0.39 [0.35–0.42], and $0 [0–0], respectively (Figure 4 and Supplementary Table S7). The consistency for LLM-assisted citation screening (at the 95% CI) using GPT-4o, Gemini 1.5 Pro, Claude 3.5 Sonnet, and Llama 3.3 70B were 98.9% [95.9–99.2], 97.8% [96.6–98.7], 95.9% [95.6–96.6], and 98.0% [97.0–98.5], respectively (Figure 4, Supplementary Tables S4 and S5).

### 3.5 Post hoc analysis of our secondary analysis using an LLM ensemble method

**Figure 5:**
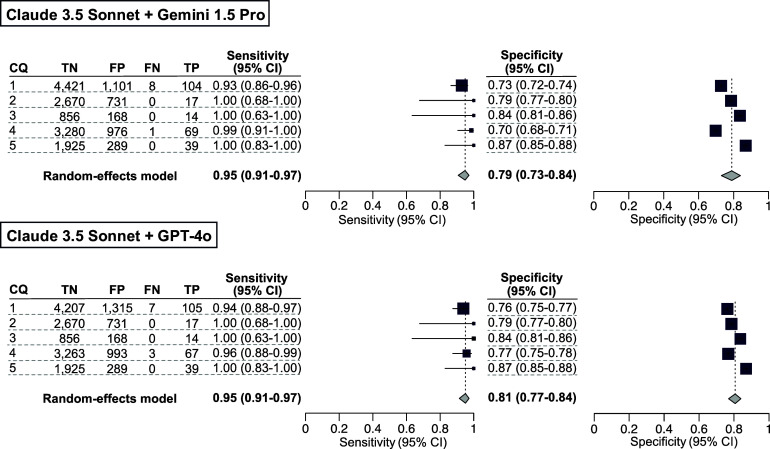
Post hoc analysis for our secondary analysis using an ensemble method. The results for the included publications were qualitatively analyzed using the conventional method as the standard reference. As a large language model ensemble method, publications included by either of the LLMs were counted as included publications. The individual sensitivity and specificity values for each clinical question (CQ), as well as the integrated sensitivity values across CQs 1–5, are presented using confidence intervals.

To enhance the sensitivity of our secondary analysis, we conducted a *post hoc* analysis by integrating the results of the three LLM-assisted citation screenings. The sensitivity and specificity values of the integrated results from Claude 3.5 Sonnet and Gemini 1.5 Pro in CQ4 were 0.99 [0.91–1.00] and 0.70 [0.68–0.71], respectively. The sensitivity and specificity values of the integrated results from Claude 3.5 Sonnet and GPT-4o in CQ1 were 0.94 [0.88–0.97] and 0.76 [0.75–0.77], respectively (Figure 5). The number of true-positive, true-negative, false-positive, and false-negative results are listed in Supplementary Table S8.

## 4 Discussion

In this study, we found that different LLMs exhibited varying levels of sensitivity and specificity across five CQs. Gemini 1.5 Pro and Claude 3.5 Sonnet demonstrated sufficiently high sensitivity (0.94) but had lower specificity (0.80–0.85), whereas GPT-4o and Llama 3.3 70B showed lower sensitivity (0.85–0.88) and higher specificity (0.93–0.97) in the primary analysis. While GPT-4o had the shortest screening time, the citation screening costs were comparable among the three models, except for Llama 3.3 70B, which incurred no charges. Furthermore, all of the models demonstrated high consistency rates, with only minor variations between them. Implementing an LLM ensemble method that integrated the results of each model enhanced sensitivity but decreased specificity.

Our analysis revealed notable differences in the performances of GPT-4o, Gemini 1.5 Pro, Claude 3.5 Sonnet, and Llama 3.3 70B, with each model exhibiting unique strengths and weaknesses regarding the citation screening process. The performance of GPT-4o and Llama 3.3 70B highlight its ability to effectively minimize false positives, which represents a critical advantage in terms of reducing workload during the initial screening phases. Even with the potential for some false negatives, a rapid scan using an LLM can be highly valuable in time-critical situations. By contrast, Gemini 1.5 Pro and Claude 3.5 Sonnet offered higher sensitivity, indicating their utility in environments where capturing every potentially relevant study is paramount. Although this increased sensitivity comes at the cost of a higher number of false positives, the corresponding reduction in false negatives provides a significant advantage by minimizing the risk of missing potentially valuable literature. Previous studies have demonstrated that the performance of several LLMs in screening citations varies widely. Among them, GPT-4 achieved the highest accuracy compared with other models available at the time of the research.^28^ ^,^ ^38^ The variations in accuracy among the models may be attributable to differences in their training datasets and algorithmic approaches,^39^ ^,^ ^40^ which can affect how each model interprets and classifies references. While LLM-assisted citation screening can serve as a *de facto* second reviewer preceding full-text screening, different performance scores highlight the necessity for systematic reviewers to consider their specific needs regarding accuracy versus workload reduction when selecting an LLM to assist their review process.

In a previous study, we confirmed the high specificity of GPT-4 Turbo for this application but were unable to achieve a satisfactory sensitivity.^25^ In this study, LLM-assisted citation screenings using Gemini 1.5 Pro and Claude 3.5 Sonnet achieved superior performance, with a sensitivity of 1.00 in each CQ but reduced specificity compared to GPT-4o and Llama 3.3 70B. Previous studies on the accuracy of LLM-based citation screening have reported sensitivities ranging from 0.32 to 1.00 and specificities ranging from 0.26 to 1.00.^24^ ^-^ ^26^ ^,^ ^28^ ^,^ ^38^ These findings highlight the variable performance of different LLMs, with newer models consistently outperforming their predecessors. Similarly, semi-automated approaches using machine learning have achieved comparable performance in prior studies, with reported sensitivities ranging from 0.75 to 0.90 and specificities ranging from 0.19 to 0.90.^41^ ^,^ ^42^ In particular, active learning techniques, which incorporate additional training data from human reviewers, have been shown to enhance accuracy.^15^ ^,^ ^17^ While machine learning methods for citation screening offer usability advantages, the variability in performance metrics implicates the need for optimizing their implementation. As few previous studies have reported such high accuracy in citation screening, or compared the performances of different LLMs for this application,^24^ ^,^ ^26^ ^,^ ^27^ ^,^ ^43^ our study emphasizes the enhanced efficiency and unique characteristics of these models for LLM-assisted citation screening.

The primary advantages of using an automated approach in the citation screening process for systematic reviews are enhanced time-related efficiency and significant workload reduction. According to our previous study, systematic review workloads can be reduced by >90% by the use of LLMs.^25^ In this study, our results indicate that GPT-4o outperformed Gemini 1.5 Pro, Claude 3.5 Sonnet, and Llama 3.3 70B in terms of processing time. GPT-4o was the most efficient LLM in terms of speed, although it had the lowest sensitivity among the four. The higher specificity of GPT-4o indicates that the overall processing time can be shortened by reducing the number of full-text reviews following the title and abstract sessions. As some relevant studies may be missed by GPT-4o, reviewers should select appropriate LLMs based on their particular priorities, balancing the need to identify potentially relevant studies with the benefit of reducing the workload for human reviewers. While our study used the character user interface (UI) for integrating the API to screen citations, we recognize that many users may prefer to use the graphical UI for its accessibility and ease of use. While semi-automatic methods can be more cost-effective over time due to their user-friendly interfaces and easier learning curve, the LLM-based approach may have a higher initial cost, particularly for users unfamiliar with coding or the technical setup required for API integration. The fundamental algorithms and models underlying the API and UI are typically the same, suggesting that the results should be broadly comparable. However, differences in features, user interactions, or potential optimizations in the UI could introduce variations in performance or user experience.

Cost can significantly influence users’ choice of LLMs for citation screening. In this context, Llama 3.3 70B, an open-source model, was identified as the most cost-effective option among the LLMs tested in this study, demonstrating better sensitivity than GPT-4o and higher specificity than Gemini 1.5 Pro and Claude 3.5 Sonnet. In contrast, closed-source commercial models present notable challenges, including limited transparency about their training data and the potential for perverse incentives that could result in misleading claims about their capabilities.^44^ The advantages of open-source models extend beyond cost-effectiveness, offering the flexibility to customize the LLM to suit specific user requirements.^45^ These aspects should be carefully considered when selecting the optimal LLM for citation screening.

Consistency in citation screening is paramount to ensuring the reliability of systematic reviews. In this study, each LLM maintained a similarly high consistency rate without significant variation. In particular, we identified few discrepancies in the studies included during our primary analysis, indicating that LLM-assisted citation screening achieves high reproducibility. While our results demonstrated that LLMs have reliability in handling the same dataset under identical conditions, variability in dataset composition may lead to differences in sensitivity and specificity. These considerations underline the need for careful dataset selection and testing across diverse scenarios. All of the models were determined to be reliable, which is critical for systematic reviews in which consistency can directly influence the conclusions drawn.

To enhance the sensitivity of the citation screening process, our *post hoc* analysis incorporated an LLM ensemble method using integrated results from the three models.^37^ This approach resulted in a slight improvement in sensitivity, at the expense of specificity. The enhanced sensitivity suggests that the LLM ensemble method can be particularly beneficial in the early stages of a systematic review when capturing a broad scope of literature is more critical than the precision of the selected studies. However, because the improvement in accuracy was small, the increased time and cost of using this method should be considered on an efficiency basis. Given that there are still many false positives, a combination of LLMs followed by active learning could be a promising approach to overcome the shortcomings of LLM-assisted citation screening.^46^ The ensemble strategy underscores the importance of tailored approaches to citation screening in which the choice of method should be aligned with the specific goals and resources of the research project.

### 4.1 Limitations

Although this study provided valuable insights into the efficiency of LLMs in systematic reviews, it was also subject to several limitations worth noting. First, we only used data from the J-SSCG 2024, which focuses on sepsis. This may have limited the generalizability of our findings to other fields. Further validation using larger and more diverse datasets would strengthen the robustness of our conclusions. Second, our metrics for assessing accuracy relied on conventional screening methods, which can vary between reviewers. Noisy labels due to variable input from human raters can influence the performance of machine-aided labeling tests.^47^ Additionally, accuracy might depend on the characteristics of the datasets.^15^ ^,^ ^16^ These considerations highlight the importance of interpreting the current findings with caution. Third, the prompt command used in this study was developed for citation screening using GPT-4 Turbo. Since different prompts exhibit varying impacts across models,^48^ our approach may have influenced the screening accuracy. However, Gemini 1.5 Pro, Claude 3.5 Sonnet, and Llama 3.3 70B exhibited superior sensitivity compared with GPT-4o—indicating that our preliminary training using GPT-4 to develop the command prompt may have ultimately had a negligible effect on the LLM comparisons. Fourth, results such as these will likely change over time as LLMs are updated and improved, potentially altering their citation screening performance. Fifth, the potential risk of cross-contamination between LLMs and the training data used by model developers remains a concern, particularly regarding the J-SSCG 2024 dataset. Specifically, we cannot rule out the possibility that the content of the guidelines and citation screening data was included in the model’s training data. Therefore, continuous monitoring is needed to address concerns about the potential inclusion of published content in LLM training. Future research should focus on validating the accuracy of LLM-assisted citation screening in other medical domains, refining these models to enhance their specificity without compromising sensitivity, and exploring their integration into other components of systematic review processes such as data extraction and meta-analysis.

## 5 Conclusions

For LLM-assisted citation screening, Gemini 1.5 Pro and Claude 3.5 Sonnet achieved satisfactory sensitivity values of 0.94, albeit with low specificity. By contrast, GPT-4o and Llama 3.3 70B demonstrated sensitivity values between 0.85 and 0.88 and higher specificity levels between 0.93 and 0.97. The screening process can be optimized based on the specific strengths of each model, considering variabilities in sensitivity, specificity, and processing times across the models.

## Supporting information

Oami et al. supplementary material Oami et al. supplementary material

## Data Availability

The datasets used and analyzed in this study are stored in the following public repository: https://github.com/seveneleven711thanks39/llm-assisted_citation_screening.git. The data files can be accessed using the password “oami2025.”

## Supplementary material

To view supplementary material for this article, please visit http://doi.org/10.1017/rsm.2025.10014.

## Clinical trial registration

The study was registered in the University Hospital Medical Information Network (UMIN) clinical trials registry (UMIN000054783).
