# Supplementary material for: Optimal large language models to screen citations for systematic reviews
Source: Res Synth Methods. 2025 Jun 23;16(6):859–75. doi: 10.1017/rsm.2025.10014 (PMC12657656; doi:10.1017/rsm.2025.10014)
Supplement: Oami et al. supplementary material [file S1759287925100148sup001.docx]

**Supplementary Online Content**

**Optimal large language models to screen citations for systematic reviews**

**Authors:** Takehiko Oami, M.D., Ph.D.^1^, Yohei Okada, M.D., Ph.D.^2, 3^, Taka-aki Nakada, M.D., Ph.D.^1^

**Affiliation**s**:**

1. Department of Emergency and Critical Care Medicine, Chiba University Graduate School of Medicine, Chiba, Japan

2. Department of Preventive Services, Kyoto University Graduate School of Medicine, Kyoto, Japan

3. Pre-hospital and Emergency Research Centre, Health Services and Systems Research, Duke-NUS Medical school, National University of Singapore, Singapore

**eAppendix.** Supplementary appendix

**eTable 1. Characteristics of the reviewers in the conventional citation screening**

**eTable 2. Statistics on the accuracy of large language model-assisted citation　screening in the primary analysis**

**eTable 3. Statistics on the accuracy of large language model-assisted citation　screening in the secondary analysis**

**eTable 4.** **Comparison of sensitivity and specificity among the four large language models in the primary analysis**

**eTable 5.** **Comparison of sensitivity and specificity among the four large language models in the secondary analysis**

**eTable 6.** **Comparison of citation screening time for 100 studies among the four large language models**

**eTable 7. Comparison of citation screening cost for 100 studies among the four large language models**

**eTable 8. Statistics on the accuracy of an ensemble method in the *post hoc* analysis for our secondary analysis (first round)**

**eAppendix**

**1. Deviations from the pre-registered protocol**

**A. Citation screening using Llama 3.3 70B**

We added citation screening using Llama 3.3 70B, an open-source model, into our evaluation.

**B. LLM ensemble method**

We performed an “LLM ensemble” method, examining the impact of combining the results from the four LLMs to potentially increase the accuracy of the LLM-assisted citation screening process as a *post hoc* analysis.

**eTable 1. Characteristics of the reviewers in the conventional citation screening**

|  | Number of reviewers (n = 18) |
| --- | --- |
| Age, years | 38.0 (35-39) |
| Male sex, n (%) | 17 (94.4) |
| Profession |  |
| Medical doctor, n (%) | 17 (94.4) |
| Nurse, n (%) | 1 (5.6) |
| Specialty^a^ |  |
| Emergency medicine, n (%) | 12 (66.7) |
| Critical care, n (%) | 14 (77.8) |
| Anesthesiology, n (%) | 4 (22.2) |
| Others | 3 (16.7) |
| Doctor of Philosophy, n (%) | 7 (38.9) |
| Years of clinical experience |  |
| 0-5 years, n (%) | 0 (0) |
| 6-10 years, n (%) | 5 (27.8) |
| 11-15 years, n (%) | 9 (50.0) |
| 16-20 years, n (%) | 4 (22.2) |
| ≥ 21 years, n (%) | 0 (0) |
| Total number of publications for systematic review |  |
| None, n (%) | 15 (83.3) |
| 1-5 papers, n (%) | 3 (16.7) |
| 6-10 papers, n (%) | 0 (0) |
| ≥ 11 papers, n (%) | 0 (0) |

Data are presented as median and interquartile range for continuous variables.

^a^ The category of specialty includes multiple inputs.

**eTable 2. Statistics on the accuracy of large language model-assisted citation　screening in the primary analysis^a^**

**A. GPT-4o**

|  | TN | FP | FN | TP |
| --- | --- | --- | --- | --- |
| First round | | | | |
| CQ1 | 5,511 | 115 | 2 | 6 |
| CQ2 | 3,234 | 181 | 1 | 3 |
| CQ3 | 1,015 | 19 | 0 | 4 |
| CQ4 | 4,058 | 251 | 0 | 17 |
| CQ5 | 2,176 | 69 | 1 | 7 |
| Second round | | | | |
| CQ1 | 5,517 | 109 | 2 | 6 |
| CQ2 | 3,233 | 181 | 1 | 3 |
| CQ3 | 1,013 | 21 | 1 | 3 |
| CQ4 | 4,047 | 262 | 0 | 17 |
| CQ5 | 2,172 | 73 | 1 | 7 |
| Third round | | | | |
| CQ1 | 5,512 | 114 | 2 | 6 |
| CQ2 | 3,236 | 178 | 1 | 3 |
| CQ3 | 1,014 | 20 | 1 | 3 |
| CQ4 | 4,059 | 250 | 0 | 17 |
| CQ5 | 2,176 | 69 | 0 | 8 |

**B. Gemini 1.5 Pro**

|  | TN | FP | FN | TP |
| --- | --- | --- | --- | --- |
| First round | | | | |
| CQ1 | 4,835 | 791 | 0 | 8 |
| CQ2 | 2,579 | 835 | 0 | 4 |
| CQ3 | 923 | 111 | 0 | 4 |
| CQ4 | 3,380 | 929 | 0 | 17 |
| CQ5 | 2,013 | 232 | 0 | 8 |
| Second round | | | | |
| CQ1 | 4,822 | 804 | 0 | 8 |
| CQ2 | 2,573 | 841 | 0 | 4 |
| CQ3 | 927 | 107 | 0 | 4 |
| CQ4 | 3,378 | 931 | 0 | 17 |
| CQ5 | 2,025 | 220 | 0 | 8 |
| Third round | | | | |
| CQ1 | 4,826 | 800 | 0 | 8 |
| CQ2 | 2,581 | 833 | 0 | 4 |
| CQ3 | 924 | 110 | 0 | 4 |
| CQ4 | 3,361 | 948 | 0 | 17 |
| CQ5 | 2,018 | 227 | 0 | 8 |

**C. Claude 3.5 Sonnet**

|  | TN | FP | FN | TP |
| --- | --- | --- | --- | --- |
| First round | | | | |
| CQ1 | 4,429 | 1197 | 0 | 8 |
| CQ2 | 2,670 | 744 | 0 | 4 |
| CQ3 | 856 | 178 | 0 | 4 |
| CQ4 | 3,281 | 1028 | 0 | 17 |
| CQ5 | 1,925 | 320 | 0 | 8 |
| Second round | | | | |
| CQ1 | 4,412 | 1214 | 0 | 8 |
| CQ2 | 2,679 | 735 | 0 | 4 |
| CQ3 | 852 | 182 | 0 | 4 |
| CQ4 | 3,273 | 1036 | 0 | 17 |
| CQ5 | 1,922 | 323 | 0 | 8 |
| Third round | | | | |
| CQ1 | 4,439 | 1187 | 0 | 8 |
| CQ2 | 2,676 | 738 | 0 | 4 |
| CQ3 | 850 | 184 | 0 | 4 |
| CQ4 | 3,268 | 1041 | 0 | 17 |
| CQ5 | 1,924 | 321 | 0 | 8 |

**D. Llama 3.3 70B**

|  | TN | FP | FN | TP |
| --- | --- | --- | --- | --- |
| First round | | | | |
| CQ1 | 5,348 | 278 | 2 | 6 |
| CQ2 | 3,233 | 181 | 0 | 4 |
| CQ3 | 969 | 65 | 0 | 4 |
| CQ4 | 3,582 | 727 | 0 | 17 |
| CQ5 | 2,122 | 123 | 0 | 8 |
| Second round | | | | |
| CQ1 | 5,338 | 288 | 2 | 6 |
| CQ2 | 3,230 | 184 | 0 | 4 |
| CQ3 | 970 | 64 | 0 | 4 |
| CQ4 | 3,574 | 735 | 0 | 17 |
| CQ5 | 2,123 | 122 | 0 | 8 |
| Third round | | | | |
| CQ1 | 5,343 | 283 | 2 | 6 |
| CQ2 | 3,239 | 175 | 0 | 4 |
| CQ3 | 972 | 62 | 0 | 4 |
| CQ4 | 3,570 | 739 | 0 | 17 |
| CQ5 | 2,126 | 119 | 0 | 8 |

CQ, clinical question; FN, false negative; FP, false positive; TN, true negative; TP, true positive

^a^ The list of included studies for qualitative analysis using the conventional method was set as the standard reference.

**eTable 3. Statistics on the accuracy of large language model-assisted citation　screening in the secondary analysis^a^**

**A. GPT-4o**

|  | TN | FP | FN | TP |
| --- | --- | --- | --- | --- |
| First round | | | | |
| CQ1 | 5,522 | 70 | 61 | 51 |
| CQ2 | 3,234 | 170 | 3 | 14 |
| CQ3 | 1,012 | 12 | 3 | 11 |
| CQ4 | 4,042 | 214 | 16 | 54 |
| CQ5 | 2,172 | 42 | 5 | 34 |
| Second round | | | | |
| CQ1 | 5,456 | 66 | 63 | 49 |
| CQ2 | 3,231 | 170 | 3 | 14 |
| CQ3 | 1,010 | 14 | 4 | 10 |
| CQ4 | 4,032 | 224 | 15 | 55 |
| CQ5 | 2,168 | 46 | 5 | 34 |
| Third round | | | | |
| CQ1 | 5,454 | 68 | 60 | 52 |
| CQ2 | 3,234 | 167 | 3 | 14 |
| CQ3 | 1,011 | 13 | 4 | 10 |
| CQ4 | 4,045 | 211 | 14 | 56 |
| CQ5 | 2,173 | 41 | 3 | 36 |

**B. Gemini 1.5 Pro**

|  | TN | FP | FN | TP |
| --- | --- | --- | --- | --- |
| First round | | | | |
| CQ1 | 4,823 | 699 | 12 | 100 |
| CQ2 | 2,579 | 822 | 0 | 17 |
| CQ3 | 922 | 102 | 1 | 13 |
| CQ4 | 3,377 | 879 | 3 | 67 |
| CQ5 | 2,012 | 202 | 1 | 38 |
| Second round | | | | |
| CQ1 | 4,811 | 711 | 11 | 101 |
| CQ2 | 2,573 | 828 | 0 | 17 |
| CQ3 | 926 | 98 | 1 | 13 |
| CQ4 | 3,375 | 881 | 3 | 67 |
| CQ5 | 2,024 | 190 | 1 | 38 |
| Third round | | | | |
| CQ1 | 4,816 | 706 | 10 | 102 |
| CQ2 | 2,581 | 820 | 0 | 17 |
| CQ3 | 923 | 101 | 1 | 13 |
| CQ4 | 3,358 | 898 | 3 | 67 |
| CQ5 | 2,018 | 196 | 0 | 39 |

**C. Claude 3.5 Sonnet**

|  | TN | FP | FN | TP |
| --- | --- | --- | --- | --- |
| First round | | | | |
| CQ1 | 4,421 | 1101 | 8 | 104 |
| CQ2 | 2,670 | 731 | 0 | 17 |
| CQ3 | 856 | 168 | 0 | 14 |
| CQ4 | 3,278 | 978 | 3 | 67 |
| CQ5 | 1,925 | 289 | 0 | 39 |
| Second round | | | | |
| CQ1 | 4,406 | 1116 | 6 | 106 |
| CQ2 | 2,679 | 722 | 0 | 17 |
| CQ3 | 852 | 172 | 0 | 14 |
| CQ4 | 3,270 | 986 | 3 | 67 |
| CQ5 | 1,922 | 292 | 0 | 39 |
| Third round | | | | |
| CQ1 | 4,432 | 1090 | 7 | 105 |
| CQ2 | 2,676 | 725 | 0 | 17 |
| CQ3 | 850 | 174 | 0 | 14 |
| CQ4 | 3,265 | 991 | 3 | 67 |
| CQ5 | 1,924 | 290 | 0 | 39 |

**D. Llama 3.3 70B**

|  | TN | FP | FN | TP |
| --- | --- | --- | --- | --- |
| First round | | | | |
| CQ1 | 5,304 | 218 | 46 | 66 |
| CQ2 | 3,233 | 168 | 0 | 17 |
| CQ3 | 968 | 56 | 1 | 13 |
| CQ4 | 3,574 | 682 | 8 | 62 |
| CQ5 | 2,120 | 94 | 2 | 37 |
| Second round | | | | |
| CQ1 | 5,297 | 225 | 43 | 69 |
| CQ2 | 3,230 | 171 | 0 | 17 |
| CQ3 | 968 | 56 | 2 | 12 |
| CQ4 | 3,566 | 690 | 8 | 62 |
| CQ5 | 2,121 | 93 | 2 | 37 |
| Third round | | | | |
| CQ1 | 5,303 | 219 | 42 | 70 |
| CQ2 | 3,239 | 162 | 0 | 17 |
| CQ3 | 971 | 53 | 1 | 13 |
| CQ4 | 3,563 | 693 | 7 | 63 |
| CQ5 | 2,124 | 90 | 2 | 37 |

CQ, clinical question; FN, false negative; FP, false positive; TN, true negative; TP, true positive

^a^ The list of included studies after title/abstract screening using the conventional method was set as the standard reference.

**eTable 4. Comparison of sensitivity and specificity among the four large language models in the primary analysis**

**A. GPT-4o**

|  | First round | | Second round | | Third round | |
| --- | --- | --- | --- | --- | --- | --- |
|  | Sensitivity | Specificity | Sensitivity | Specificity | Sensitivity | Specificity |
| CQ1 | 0.75 | 0.98 | 0.75 | 0.98 | 0.75 | 0.98 |
| CQ2 | 0.75 | 0.95 | 0.75 | 0.95 | 0.75 | 0.95 |
| CQ3 | 1.00 | 0.98 | 0.75 | 0.98 | 0.75 | 0.98 |
| CQ4 | 1.00 | 0.94 | 1.00 | 0.94 | 1.00 | 0.94 |
| CQ5 | 0.88 | 0.97 | 0.88 | 0.97 | 1.00 | 0.97 |

**B. Gemini 1.5 Pro**

|  | First round | | Second round | | Third round | |
| --- | --- | --- | --- | --- | --- | --- |
|  | Sensitivity | Specificity | Sensitivity | Specificity | Sensitivity | Specificity |
| CQ1 | 1.00 | 0.86 | 1.00 | 0.86 | 1.00 | 0.86 |
| CQ2 | 1.00 | 0.76 | 1.00 | 0.75 | 1.00 | 0.76 |
| CQ3 | 1.00 | 0.89 | 1.00 | 0.90 | 1.00 | 0.89 |
| CQ4 | 1.00 | 0.78 | 1.00 | 0.78 | 1.00 | 0.78 |
| CQ5 | 1.00 | 0.90 | 1.00 | 0.90 | 1.00 | 0.90 |

**C. Claude 3.5 Sonnet**

|  | First round | | Second round | | Third round | |
| --- | --- | --- | --- | --- | --- | --- |
|  | Sensitivity | Specificity | Sensitivity | Specificity | Sensitivity | Specificity |
| CQ1 | 1.00 | 0.79 | 1.00 | 0.78 | 1.00 | 0.79 |
| CQ2 | 1.00 | 0.78 | 1.00 | 0.78 | 1.00 | 0.78 |
| CQ3 | 1.00 | 0.83 | 1.00 | 0.82 | 1.00 | 0.82 |
| CQ4 | 1.00 | 0.76 | 1.00 | 0.76 | 1.00 | 0.76 |
| CQ5 | 1.00 | 0.86 | 1.00 | 0.86 | 1.00 | 0.86 |

**D. Llama 3.3 70B**

|  | First round | | Second round | | Third round | |
| --- | --- | --- | --- | --- | --- | --- |
|  | Sensitivity | Specificity | Sensitivity | Specificity | Sensitivity | Specificity |
| CQ1 | 0.75 | 0.95 | 0.75 | 0.95 | 0.75 | 0.95 |
| CQ2 | 1.00 | 0.95 | 1.00 | 0.95 | 1.00 | 0.95 |
| CQ3 | 1.00 | 0.94 | 1.00 | 0.94 | 1.00 | 0.89 |
| CQ4 | 1.00 | 0.83 | 1.00 | 0.83 | 1.00 | 0.83 |
| CQ5 | 1.00 | 0.95 | 1.00 | 0.95 | 1.00 | 0.95 |

LLM: large language model, CQ: clinical question

**eTable 5. Comparison of sensitivity and specificity among the four large language models in the secondary analysis**

**A. GPT-4o**

|  | First round | | Second round | | Third round | |
| --- | --- | --- | --- | --- | --- | --- |
|  | Sensitivity | Specificity | Sensitivity | Specificity | Sensitivity | Specificity |
| CQ1 | 0.46 | 0.99 | 0.44 | 0.99 | 0.46 | 0.99 |
| CQ2 | 0.82 | 0.95 | 0.82 | 0.95 | 0.82 | 0.95 |
| CQ3 | 0.79 | 0.99 | 0.71 | 0.99 | 0.71 | 0.99 |
| CQ4 | 0.77 | 0.95 | 0.79 | 0.95 | 0.80 | 0.95 |
| CQ5 | 0.87 | 0.98 | 0.87 | 0.98 | 0.92 | 0.98 |

**B. Gemini 1.5 Pro**

|  | First round | | Second round | | Third round | |
| --- | --- | --- | --- | --- | --- | --- |
|  | Sensitivity | Specificity | Sensitivity | Specificity | Sensitivity | Specificity |
| CQ1 | 0.89 | 0.87 | 0.90 | 0.87 | 0.91 | 0.87 |
| CQ2 | 1.00 | 0.76 | 1.00 | 0.76 | 1.00 | 0.76 |
| CQ3 | 0.93 | 0.90 | 0.93 | 0.90 | 0.93 | 0.90 |
| CQ4 | 0.96 | 0.79 | 0.96 | 0.79 | 0.96 | 0.79 |
| CQ5 | 0.97 | 0.91 | 0.97 | 0.91 | 1.00 | 0.91 |

**C. Claude 3.5 Sonnet**

|  | First round | | Second round | | Third round | |
| --- | --- | --- | --- | --- | --- | --- |
|  | Sensitivity | Specificity | Sensitivity | Specificity | Sensitivity | Specificity |
| CQ1 | 0.93 | 0.80 | 0.95 | 0.80 | 0.94 | 0.80 |
| CQ2 | 1.00 | 0.79 | 1.00 | 0.79 | 1.00 | 0.79 |
| CQ3 | 1.00 | 0.84 | 1.00 | 0.83 | 1.00 | 0.83 |
| CQ4 | 0.96 | 0.77 | 0.96 | 0.77 | 0.96 | 0.77 |
| CQ5 | 1.00 | 0.87 | 1.00 | 0.87 | 1.00 | 0.87 |

**D. Llama 3.3 70B**

|  | First round | | Second round | | Third round | |
| --- | --- | --- | --- | --- | --- | --- |
|  | Sensitivity | Specificity | Sensitivity | Specificity | Sensitivity | Specificity |
| CQ1 | 0.59 | 0.96 | 0.62 | 0.96 | 0.63 | 0.96 |
| CQ2 | 1.00 | 0.95 | 1.00 | 0.95 | 1.00 | 0.95 |
| CQ3 | 0.93 | 0.95 | 0.86 | 0.95 | 0.93 | 0.95 |
| CQ4 | 0.89 | 0.84 | 0.89 | 0.84 | 0.90 | 0.84 |
| CQ5 | 0.95 | 0.96 | 0.95 | 0.96 | 0.95 | 0.96 |

LLM: large language model, CQ: clinical question

**eTable 6. Comparison of citation screening time for 100 studies among the four large language models**

**A. GPT-4o**

|  | First round | Second round | Third round |
| --- | --- | --- | --- |
|  | Time (min) | Time (min) | Time (min) |
| CQ1 | 1.0 | 1.0 | 1.1 |
| CQ2 | 0.9 | 0.9 | 0.9 |
| CQ3 | 0.9 | 1.0 | 0.9 |
| CQ4 | 0.9 | 0.9 | 1.0 |
| CQ5 | 0.9 | 1.0 | 0.9 |

**B. Gemini 1.5 Pro**

|  | First round | Second round | Third round |
| --- | --- | --- | --- |
|  | Time (min) | Time (min) | Time (min) |
| CQ1 | 1.5 | 1.5 | 1.6 |
| CQ2 | 1.8 | 1.4 | 1.6 |
| CQ3 | 1.4 | 1.4 | 1.7 |
| CQ4 | 1.7 | 1.8 | 1.7 |
| CQ5 | 1.5 | 1.4 | 1.5 |

**C. Claude 3.5 Sonnet**

|  | First round | Second round | Third round |
| --- | --- | --- | --- |
|  | Time (min) | Time (min) | Time (min) |
| CQ1 | 3.6 | 3.7 | 3.9 |
| CQ2 | 2.7 | 3.1 | 3.0 |
| CQ3 | 2.9 | 3.5 | 3.4 |
| CQ4 | 3.4 | 4.4 | 3.7 |
| CQ5 | 3.1 | 3.4 | 3.2 |

**D. Llama 3.3 70B**

|  | First round | Second round | Third round |
| --- | --- | --- | --- |
|  | Time (min) | Time (min) | Time (min) |
| CQ1 | 1.0 | 1.3 | 1.3 |
| CQ2 | 1.3 | 1.2 | 0.9 |
| CQ3 | 1.2 | 1.2 | 1.0 |
| CQ4 | 1.1 | 1.3 | 1.2 |
| CQ5 | 1.4 | 1.4 | 1.0 |

LLM: large language model, CQ: clinical question

**eTable 7. Comparison of citation screening cost for 100 studies among the four large language models**

**A. GPT-4o**

|  | First round | Second round | Third round |
| --- | --- | --- | --- |
|  | Cost ($) | Cost ($) | Cost ($) |
| CQ1 | 0.39 | 0.40 | 0.39 |
| CQ2 | 0.40 | 0.40 | 0.40 |
| CQ3 | 0.39 | 0.27 | 0.39 |
| CQ4 | 0.42 | 0.43 | 0.42 |
| CQ5 | 0.50 | 0.51 | 0.50 |

**B. Gemini 1.5 Pro**

|  | First round | Second round | Third round |
| --- | --- | --- | --- |
|  | Cost ($) | Cost ($) | Cost ($) |
| CQ1 | 0.28 | 0.26 | 0.29 |
| CQ2 | 0.29 | 0.28 | 0.28 |
| CQ3 | 0.27 | 0.27 | 0.27 |
| CQ4 | 0.36 | 0.24 | 0.30 |
| CQ5 | 0.48 | 0.55 | 0.35 |

**C. Claude 3.5 Sonnet**

|  | First round | Second round | Third round |
| --- | --- | --- | --- |
|  | Cost ($) | Cost ($) | Cost ($) |
| CQ1 | 0.37 | 0.37 | 0.38 |
| CQ2 | 0.39 | 0.40 | 0.39 |
| CQ3 | 0.33 | 0.33 | 0.36 |
| CQ4 | 0.37 | 0.41 | 0.40 |
| CQ5 | 0.44 | 0.44 | 0.43 |

**D. Llama 3.3 70B**

|  | First round | Second round | Third round |
| --- | --- | --- | --- |
|  | Cost ($) | Cost ($) | Cost ($) |
| CQ1 | 0 | 0 | 0 |
| CQ2 | 0 | 0 | 0 |
| CQ3 | 0 | 0 | 0 |
| CQ4 | 0 | 0 | 0 |
| CQ5 | 0 | 0 | 0 |

LLM: large language model, CQ: clinical question

**eTable 8. Statistics on the accuracy of an ensemble method in the *post hoc* analysis for our secondary analysis (first round)**

**A. Claude 3.5 Sonnet + Gemini 1.5 Pro**

|  | TN | FP | FN | TP |
| --- | --- | --- | --- | --- |
| Secondary analysis^a^ | | | | |
| CQ1 | 4,421 | 1101 | 8 | 104 |
| CQ2 | 2,670 | 731 | 0 | 17 |
| CQ3 | 856 | 168 | 0 | 14 |
| CQ4 | 3,280 | 976 | 1 | 69 |
| CQ5 | 1,925 | 289 | 0 | 39 |

**B. Claude 3.5 Sonnet + GPT-4o**

|  | TN | FP | FN | TP |
| --- | --- | --- | --- | --- |
| Secondary analysis^a^ | | | | |
| CQ1 | 4,207 | 1315 | 7 | 105 |
| CQ2 | 2,670 | 731 | 0 | 17 |
| CQ3 | 856 | 168 | 0 | 14 |
| CQ4 | 3,263 | 993 | 3 | 67 |
| CQ5 | 1,925 | 289 | 0 | 39 |

CQ, clinical question; FN, false negative; FP, false positive; TN, true negative; TP, true positive

^a^ The list of included studies after title/abstract screening using the conventional method was set as the standard reference.
